# Asbestosis is prevalent in a variety of construction industry trades

**DOI:** 10.1038/s41533-018-0078-6

**Published:** 2018-04-03

**Authors:** G. I. Walters, A. S. Robertson, P. S. Bhomra, P. S. Burge

**Affiliations:** Birmingham Regional NHS Occupational Lung Disease Service, Birmingham Chest Clinic, Birmingham, UK

## Abstract

A diagnosis of asbestosis, which is a long-latency, fibrotic lung disease, has implications for the patient in terms of prognosis, treatment and compensation. Identifying and quantifying asbestos exposure is difficult without a detailed occupational history, and the threshold dose of asbestos required to cause asbestosis is not well understood. We reviewed all cases of asbestosis diagnosed between 2001 and 2016 at the Birmingham Regional NHS Occupational Lung Disease Service to determine the industries and occupations most frequently implicated in causation, in order to help clinicians identify where asbestosis might enter the differential diagnosis for a patient with chronic respiratory symptoms. A variety of construction trades were frequently reported including carpenters and joiners, pipe fitters, laggers, labourers, painters and shop fitters. Traditionally heavily exposed occupations such as shipbuilding were not commonly seen.

## Introduction

Asbestosis is a diffuse interstitial fibrosis caused by inhalational exposure to asbestos after a latent period >20 years.^1^ It is a diagnosis made through a history of exposure, exclusion of other causes and the characteristic computer tomography (CT) appearances of usual interstitial pneumonia; that is, bilateral subpleural reticulation in a basal distribution, with bronchial dilatation and honeycombing.^2^ Current National Health Service (NHS) policy prevents prescription of antifibrotics for asbestosis^3^ though workers with confirmed asbestosis are eligible for state compensation.^4^ Thus, differentiating asbestosis from idiopathic pulmonary fibrosis (IPF) through exposure history is important but can be difficult, particularly because the dose of asbestos required to cause fibrosis is unknown.^5^ Heavy exposures as seen with asbestos miners, millers and textile workers has caused severe fibrosis with relatively short latency,^6^ but our current experience is one of asbestosis with comparatively lower exposures but longer latencies from work undertaken in the 1960–1970s, before the impact of UK regulations banning the import and use of asbestos.^7^ We aimed to identify the industries and occupations in patients diagnosed with asbestosis at a UK regional NHS occupational lung disease service to help clinicians identify those in whom a full exposure history is indicated.

## Results

### Demographics

There were 160 cases of asbestosis diagnosed between 1 January 2001 and 31 December 2015 [2001–2005 (34), 2006–2010 (35), 2011–2015 (91); median = 10 cases per annum, interquartile range (IQR) = 5–13]. Of 160 patients, 158 (99%) were males and median age at diagnosis was 74 (IQR = 69–79) with age distribution as follows: 51–60 years (3%), 61–70 years (33%), 71–80 years (43%) and 81–90 years (21%). At diagnosis 41 patients (26%) had asbestosis only, 117 (73%) had additional benign asbestos pleural disease (BAPD), 2 (1%) had mesothelioma (one with BAPD), and 2 (1%) had bronchial cancer (one with BAPD).

### Industries and occupations

**Fig. 1:**
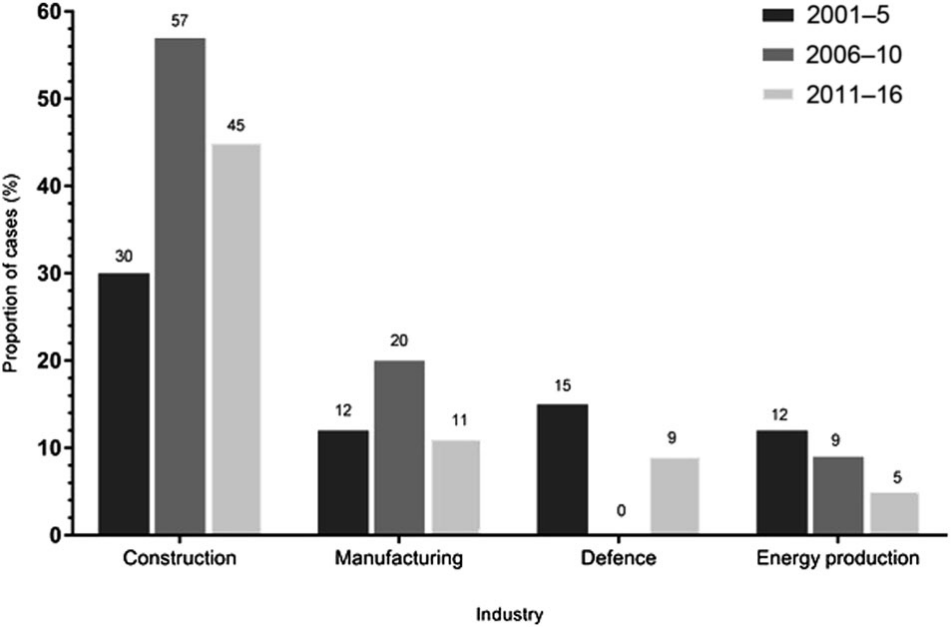
Common industries implicated in causation of asbestosis 2001–2016 at Birmingham Regional NHS Occupational Lung Disease Service. Construction was consistently the most frequently encountered industry during each 5-year period, becoming proportionally more common in 2006–2010 (*p* = 0.02) and 2011–2016 (*p* = 0.15) when compared to 2001–2005

Commonly encountered industries and occupations are shown in Table 1. Construction was the commonest industry (44%) and most frequently identified across each 5-year period, becoming proportionally more common after 2005 (Fig. 1). Construction workers were most likely to be carpenters or joiners (12; 17%), laggers or lagger’s mates (10; 14%), painters or decorators (7; 10%), shop fitters (7; 10%), roofers (5; 7%), labourers (5; 7%), pipe fitters or fitter’s mates (4; 6%), plumbers (4; 6%), plasterers (4; 6%), floorers and tilers (4; 6%), sheet metal workers (2; 3%) or other (7; 10%). The two female patients were (1) a secretary from a British-based asbestos supply company and (2) a Portuguese civil servant now living in Britain, who worked in an asbestos mine near Chimoio in Mozambique between 1968 and 1972.

**Table 1:** Cases of asbestosis (2001–2016) at Birmingham Regional NHS Occupational Lung Disease Service, showing the frequencies of encountered industries (A) and occupations (B)

	Number of cases (%)
(A) Industries	
Construction	71 (44)
Manufacturing	21 (13)
Defence	13 (8)
Energy production	12 (8)
Automotive	10 (6)
Metals	6 (4)
Shipping and shipbuilding	6 (4)
Asbestos production and supply	4 (3)
Demolition	3 (2)
Healthcare	2 (1)
Chemical	2 (1)
Education	2 (1)
Aerospace	1 (<1)
Civil service	1 (<1)
Prison service	1 (<1)
Confectionery	1 (<1)
Fishing	1 (<1)
Parks and grounds	1 (<1)
Rail	1 (<1)
Telecommunications	1 (<1)
(B) Occupations	
Pipe fitter/pipe fitter’s mate [including fitter-welders]	20 (13)
Carpenter/joiner	17 (11)
Lagger/lagger’s mate	16 (10)
Building labourer	9 (6)
Painter/decorator [includes domestic and industrial]	8 (5)
Engineer [includes metal, civil, electrical, railway, naval]	8 (5)
Steel fabricator-welder/ steel erector/welder’s mate	7 (4)
Carriage fitter	6 (4)
Boiler man/stoker	6 (4)
Roofer	5 (3)
Mechanic [includes rail, car, military]	5 (3)
Plumber	5 (3)
Electrician	5 (3)
Sheet metal worker	4 (3)
Plasterer	4 (3)
Floorer/tiler	4 (3)
Driver [including trains and excavators]	4 (3)
Scientist/laboratory technician	3 (2)
Assembler	3 (2)
Inspector	2 (1)
Manager	2 (1)
Asbestos mixer	2 (1)
Shop fitter	2 (1)
Other [single entries]	13 (8)

Manufacturing included production of train carriages (9 patients), cookers and ovens, refrigerators, plastics, shoes, tyres, ironing boards, electrical items and table sauce; the nine patients from train carriage manufacturing were from a single employer and were: carriage fitters (6), carpenter (1), painter (1) and sheet metal worker (1). Those employed by the Ministry of Defence (defence industry) were naval shipwrights/fitters and electricians (10), articifer (1), railway mechanic (1) and metal worker (1). Of 12 patients, 9 employed in the energy industry worked in power stations as fitter-welders (5), laggers or lagger’s mates (3) and a steel erector (1).

## Discussion

A variety of trades within the construction industry were commonly responsible for exposure sufficient to cause asbestosis: particularly carpenters and joiners, laggers, painters and shop fitters. Pipe fitters, labourers, steel workers and welders were also frequently implicated, seen in a number industries (including construction). Work in naval engineering, train carriage manufacture and in power stations were also regularly encountered, but other traditionally heavily exposed UK occupations such as shipbuilding were not seen commonly.

Asbestosis was first described in the UK in 1924 in a 33-year-old female asbestos factory worker.^8^ Although further health risks of asbestos subsequently emerged, UK asbestos consumption increased throughout the twentieth century.^9^ Its properties of low cost, strength, flexibility and resistance to chemical corrosion, heat and fire made it desirable, particularly during World War II and the construction boom that followed. Consumption in the UK reached a peak in the 1960s, though the highest risk of asbestosis is observed 40–60 years after first exposure;^10^ thus annual deaths and compensated cases have continued to increase year on year since 1978.^11^ In the UK, insulation workers followed by asbestos stripping/removal workers have the highest risk of asbestosis, followed by shipbuilders and construction workers.^10^

In this study, it is not possible to infer and quantify risk from employment without knowing when and how much exposure occurred or without population figures for the number of exposed workers at the time. Moreover, an increase in reported cases does not necessarily imply an increase in incidence of asbestosis, and other factors such as better and more frequent CT imaging, and the evolution of multi-disciplinary team decision making (i.e. physician, thoracic radiologist, etc.) may contribute to this. This is the experience of one regional UK centre and employment characteristics in other regions may vary, for example, one might expect to see more shipbuilders in Scotland and the North-East, where UK shipyards were concentrated, but not in the West Midlands. Indeed historically the West Midlands has seen lower rates of asbestosis than average for England, and significantly less than the North-West and North-East,^11^ suggesting different levels of exposure by region.

These results are broadly supported by studies which have shown heavy exposure and increased risk of lung cancer and mesothelioma in UK carpenters,^12,13^ thought to be due to the use of asbestos insulation board, and an increased risk in other construction trades (electricians, plumbers, pipe fitters and steel workers) elsewhere.^14^ A small number of workers in this study had co-existing thoracic malignancy at the time of diagnosis, though future risk of bronchial cancer and mesothelioma cannot be extrapolated from this data.

The advent of UK asbestos control legislation during the 1970s and beyond has resulted in lower workplace exposures to asbestos.^15,16^ The current trend of increasing incidence of asbestosis reflects the latency from previous heavy exposures. If, as the Health and Safety Executive (HSE) maintains, heavy asbestos exposures are required to cause clinically significant asbestosis during an exposed worker’s lifetime, one would predict a decline in incidence during the next decade.^10^ However, there is a strong association between asbestos imports and mortality from IPF, a disease whose incidence is rising. This is similar in magnitude to the association between asbestos imports and mesothelioma and this has raised an as yet unanswered question, about whether a proportion of IPF is actually asbestosis, caused by ‘hidden’ or low-dose asbestos exposures.^17^

Asbestosis is an important diagnosis to suspect since the natural history may be different to IPF; there is an increased risk of mesothelioma when asbestos exposure has been recognized, and workers are eligible for state compensation. It is worthwhile considering asbestosis and referral for a full occupational history in patients who have worked in construction trades, presenting with chronic respiratory symptoms.

## Methods

All cases of asbestosis diagnosed at the regional NHS Occupational Lung Disease Service in Birmingham, UK between 1 January 2001 and 31 December 2016 have been retained on a Microsoft Excel clinical database containing pseudo-anonymized data (by reference to hospital identification number only). These local data are used for monthly notifications to the UK Health and Safety Executive (HSE) national “Surveillance of Work-related Respiratory Disease (SWORD)” voluntary surveillance scheme for occupational lung disease.^18^ Diagnosis of asbestosis is based on exposure history and expert opinion on whether dose is sufficient to cause asbestosis, CT appearance and exclusion of other causes of usual interstitial pneumonia. From the database the following data were gathered, without recourse to individual patients’ medical records: age at diagnosis, gender, presence of additional asbestos-related diseases at diagnosis of asbestosis, and industry and occupation where exposure occurred. Descriptive analyses were performed; non-normally distributed data were displayed using median and IQR, and categorical data with percentages. Chi-squared testing at the 95% confidence level was used to look for significant differences between categories. The study was considered to be a service evaluation by UK Health Research Authority and Heart of England NHS Foundation Trust, and as such no ethical approval was sought.

### Data availability

The data that support the findings of this study are available from the corresponding author upon reasonable request.
